# Sores

**Published:** 1859-04

**Authors:** 


### SORES.

SOMETIME ago, a little child had a pimple on its breast, which became a little sore, an amateur doctor advised a "simple" remedy to be applied to it, which was done, neither the advice nor the remedy cost anything, except the child's life in forty-eight hours.

A gentleman had a small running sore on the top of his foot; he was anxious to have it "cured up," we advised him to keep it running, but that was troublesome, and it was healed up, in a short time, a cold set in, and he died of consumption, at the end of a year.

The son of a merchant wanted to reduce a swelling in the side, which began to "run;" general remedies were proposed, with advice to let the "sore" alone. This did not suit his views, so he had it healed up, and died of consumption within two years.

A lady had a sore on the leg: it interfered with her walking, she was impatient to have it "cured up," but was advised as to the consequences, but this was disregarded. The sore was healed up, and she died of consumption.

A lady aged seventy had a sore leg, and was extremely anxious to have it healed up. She was advised by all means to make no application to it, but merely to keep it under control by general remedies. An old woman was applied to, and with various salves a wonderful "cure" was the result. Within a few weeks the lady took dinner in usual health, and died in an hour.

A lady aged sixty six had a long continued violent and painful cough, a "running" took place under the toe nail, she feared mortification, and was anxious to have it healed up. She was told that it was the best thing that could have happened to her, inasmuch as it would probably cure her cough, and add years to her life, while by improving the general health the running might slowly dry up of itself, all which proved to be so, and now in her seventy-fourth year, she has better health than half our women at forty.

Our object in stating these facts which have occured in our own experience, within a comparatively short time, is to impress upon the reader's mind, the signal danger of tampering with sores, especially such as are sometimes called "old sores,"

for they are the outlet to disease, and if injudiciously closed, what they would have discharged, will be thrown in upon more vital internal organs, causing apoplexy, consumption or fatal congestions, as certainly as the boiler of a locomotive will be shivered to atoms, if the fire is continued, and all escape of steam is prevented. In all cases of old sores, apply to a physician of age and experience. If that is not practicable, the safest and best plan is first, to diminish the amount of food eaten each day, one half, and keep the parts in a cleanly condition, by washing them twice a day in soft, milk-warm water, until relief is given.

